# TNF-α Regulates Human Plasmacytoid Dendritic Cells by Suppressing IFN-α Production and Enhancing T Cell Activation

**DOI:** 10.4049/jimmunol.1901358

**Published:** 2021-01-13

**Authors:** Antonios Psarras, Agne Antanaviciute, Adewonuola Alase, Ian Carr, Miriam Wittmann, Paul Emery, George C. Tsokos, Edward M. Vital

**Affiliations:** * Leeds Institute of Rheumatic and Musculoskeletal Medicine, University of Leeds, Leeds LS7 4SA, United Kingdom;; † National Institute for Health Research Leeds Biomedical Research Centre, Leeds Teaching Hospitals NHS Trust, Leeds LS7 4SA, United Kingdom;; ‡ Division of Rheumatology, Beth Israel Deaconess Medical Center, Harvard Medical School, Boston, MA 02215; and; § Leeds Institute for Data Analytics, University of Leeds, Leeds LS9 7TF, United Kingdom

## Abstract

Human plasmacytoid dendritic cells (pDCs) play a vital role in modulating immune responses. They can produce massive amounts of type I IFNs in response to nucleic acids via TLRs, but they are also known to possess weak Ag-presenting properties inducing CD4^+^ T cell activation. Previous studies showed a cross-regulation between TNF-α and IFN-α, but many questions remain about the effect of TNF-α in regulating human pDCs. In this study, we showed that TNF-α significantly inhibited the secretion of IFN-α and TNF-α of TLR-stimulated pDCs. Instead, exogenous TNF-α promoted pDC maturation by upregulating costimulatory molecules and chemokine receptors such as CD80, CD86, HLA-DR, and CCR7. Additionally, RNA sequencing analysis showed that TNF-α inhibited IFN-α and TNF-α production by downregulating IRF7 and NF-κB pathways, while it promoted Ag processing and presentation pathways as well as T cell activation and differentiation. Indeed, TNF-α–treated pDCs induced in vitro higher CD4^+^ T cell proliferation and activation, enhancing the production of Th1 and Th17 cytokines. In conclusion, TNF-α favors pDC maturation by switching their main role as IFN-α–producing cells to a more conventional dendritic cell phenotype. The functional status of pDCs might therefore be strongly influenced by their overall inflammatory environment, and TNF-α might regulate IFN-α–mediated aspects of a range of autoimmune and inflammatory diseases.

## Key Points

- TNF downregulates IFN-α and TNF production by human pDCs.
- TNF downregulates IRF7 and NF-κB pathways and upregulates Ag processing in pDCs.
- TNF enhances Ag presentation and T cell activation properties in pDCs.

## Introduction

Human plasmacytoid dendritic cells (pDCs) consist of a distinct DC population that play a vital role in modulating immune responses. A common DC progenitor in the bone marrow can generate both pDCs and conventional DCs (cDCs), but pDCs are unique in their ability to produce type I IFNs in response to viral infection (1). Upon ligation of TLR7 and TLR9 with exogenous or endogenous nucleic acids, pDCs can secrete massive amounts of type I IFNs, predominantly IFN-α, and other proinflammatory cytokines. These effects lead to activation in both innate and adaptive immune compartments such as enhancement of NK cell cytotoxicity, effector CD4^+^ and CD8^+^ T cell responses, B cell differentiation into plasma cells, and Ab production (2–7).

Apart from type I IFN production, other cytokines such as TNF-α can also be produced by pDCs upon viral stimulation (3). Early studies demonstrated that the production of IFN-α, IFN-β, and TNF-α by virus-stimulated pDCs can act on an autocrine fashion on the cells, affecting their survival and further differentiation enhancing T cell–mediated antiviral immunity (3, 8). More recent transcriptomic data demonstrated that influenza can result in differentiation of pDCs into multiple subgroups with distinct phenotypes and functional properties (9).

Although not as efficient as cDCs, pDCs express MHC class II (MHC-II) molecules and are able to capture, process, and present Ags to CD4^+^ T cells, inducing their activation (10, 11). Receptors specifically found on pDCs such as BDCA-2 can play a role in Ag internalization switching the T cell activation properties of the cells (12, 13). TLR-activated pDCs have enhanced Ag-presenting function and can promote Th1 and Th17 differentiation (14–16). Despite their weaker Ag-presenting properties, pDCs can also cross-present exogenous Ags to CD8^+^ T cells and therefore induce antiviral and antitumor responses (5, 17, 18). However, unstimulated pDCs predominantly facilitate tolerogenic immune responses by expressing IDO and promoting CD4^+^ T cell anergy and regulatory T cell differentiation (19–22).

As the main drivers of type I IFN responses, pDCs have been implicated in many diseases, especially chronic viral infections, cancer, and autoimmunity (23–26). Multiple regulatory surface receptors (e.g., BDCA-2, ILT7, BST2, and NKp44) control the aberrant production of type I IFNs by TLR-activated pDCs (12, 27, 28). Cross-regulation of TNF-α and IFN-α appears to be important in many immune-mediated diseases (29–31). Previous work on pDCs generated in vitro from CD34^+^ hematopoietic progenitors clearly demonstrated a cross-regulation between TNF-α and type I IFNs (31). TNF-α was shown not only to inhibit the in vitro generation of pDCs but also to downregulate influenza-induced IFN-α production. In addition, neutralization of endogenous TNF-α secreted by influenza-stimulated pDCs could lead to partially sustained IFN-α production (31). However, the mechanism defining how TNF-α regulates these changes in IFN production and the effects of TNF on other pDC functions still remains less well understood.

In this study, we investigated the regulatory role of TNF-α on the phenotype and function of blood-purified human pDCs. We found that TNF-α is a major cytokine produced alongside IFN-α by TLR9- or TLR7-stimulated pDCs and that exogenous TNF-α strongly inhibited both IFN-α and TNF-α production, an effect which is predominantly TLR9 and less TLR7 mediated. Additionally, TNF-α induced a distinct transcriptomic profile in pDCs by promoting pathways related to Ag processing and presentation as well as enhancing the ability of pDCs to induce T cell proliferation, activation, and differentiation toward Th1 and Th17 in vitro. Our findings demonstrate that TNF-α is a major regulator of human pDCs and can enhance their function by switching their main role as IFN-α–producing cells to a more cDC phenotype.

## Materials and Methods

### Isolation of human cells

Human PBMCs were separated from whole blood by density gradient centrifugation using Leucosep tubes (Greiner Bio-One). A pDC-enriched population was isolated from PBMCs by negative selection using the Diamond pDC Isolation Kit II (Miltenyi Biotec). pDCs were further sorted using an Ab to BDCA-4 (Miltenyi Biotec). Naive CD4^+^ T cells were purified by negative selection using the Naive CD4^+^ T Cell Isolation Kit II (Miltenyi Biotec). Cell sorting was carried out at the St. James Campus Infrastructure and Facilities Cytometry and Imaging Facility of the Wellcome Trust Brenner Building of the University of Leeds, with a BD Influx Six-Way Cell Sorter (BD Biosciences).

### Cell cultures

Cells were cultured in RPMI medium 1640 with GlutaMAX supplement (Thermo Fisher Scientific) containing 10% (vol/vol) FBS and 100 U/ml penicillin/streptomycin. For cytokine production, cells were stimulated with 2 μM class A CpG (ODN 2216; Miltenyi Biotec) or ORN R-2336 (Miltenyi Biotec). For pDC/T cell coculture, pre-enriched pDCs were treated with 10 ng/ml recombinant human TNF-α (R&D Systems) for 24 h and then washed twice to remove residual TNF-α before use in subsequent culture. Untreated or TNF-α–treated pDCs were cultured with allogeneic naive CD4^+^ T cells at 1:5 ratio for 5 d. For cytokine detection, cells were cultured in the last 4 h with GolgiPlug (BD Biosciences). Cell proliferation was measured using the CellTrace Violet Cell Proliferation kit (Thermo Fisher Scientific), according to the manufacturer’s instructions.

### Human TNF-α neutralization

Pre-enriched pDCs were stimulated with ODN 2216 (1 ng/ml) or ORN R-2336 (1 ng/ml) in the presence or absence of human TNF-α Ab (R&D Systems). After 24 h, the plates were centrifuged to collect the supernatants, and the cells were washed twice before restimulation for an additional 24 h. Supernatants collected at 24 and 48 h were analyzed by Human IFN-α Platinum ELISA Kit (eBioscience), according to the manufacturer’s protocol.

### Flow cytometry

For cell surface staining, fluorochrome-conjugated mAbs against human CD3, CD4, CD19, CD14, CD56, CD11c, HLA-DR, CD123, CD303, CD317, CD80, CD85g, isotype controls (Miltenyi Biotec), CD304, CD69 (BioLegend), CD86, and CCR7 (BD Biosciences) were used. For intracellular staining, cells were first stained for surface markers and then fixed and permeabilized using Intracellular Fixation and Permeabilization Buffer Set (eBioscience) before incubation with fluorochrome-conjugated mAbs against human IFN-α and TNF-α (Miltenyi Biotec) or TNF-α, IFN-γ, and IL-17A (BioLegend). Flow cytometry was performed on LSRII (BD Biosciences) and Cytoflex S (Beckman Coulter) cytometers, and the data were analyzed using FACSDiva (BD Biosciences) and CytExpert (Beckman Coulter) software.

### Measurement of Ag uptake

pDCs were isolated from PBMCs (Miltenyi Biotec) and were then cultured in RPMI medium 1640 with GlutaMAX supplement (Thermo Fisher Scientific) containing 10% (vol/vol) FBS and 100 U/ml penicillin/streptomycin in a 96-well plate. Purified pDCs were cultured with or without 10 μg/ml DQ OVA (Molecular Probes) in the presence or absence of 50 ng/ml TNF-α. After 18 h, the cells were collected and then washed twice before data were analyzed using flow cytometry.

### RNA sequencing data generation

RNA from sorted pDCs was extracted using PicoPure RNA Isolation Kit (Thermo Fisher Scientific) and quantified using Qubit RNA HS Assay Kit (Thermo Fisher Scientific). RNA libraries were made by using SMART-Seq V4 Ultra Low Input RNA Kit (Takara Bio) and Nextera XT DNA Library Preparation Kit (Illumina) for next-generation sequencing. Indexed sequencing libraries were pooled and sequenced on a single lane on HiSeq 3000 instrument as 151 bp paired-end reads. Pooled sequence data were then demultiplexed using Illumina bcl2fastq software, allowing no mismatches in the read index sequences.

### RNA sequencing data processing and analysis

Raw paired-end sequence data in Fastq format was initially analyzed using FastQC software to identify potential issues with data quality. Cutadapt software (32) was then used to remove poor quality bases (Phred quality score <20) and contaminating technical sequences from raw sequenced reads. Contaminating technical sequences identified at the initial quality control stage were as follows: CTGTCTCTTATA, next era transposase sequence; GTATCAACGCAGAGTACT, SMART-Seq oligonucleotide sequence; and dT30, SMART-Seq 3′ CDS primer II sequence.

Reads trimmed to fewer than 30 nt and orphaned mate-pair reads were discarded to minimize alignment errors downstream.

Reads were aligned to human hg38 analysis set reference sequences, obtained from University of California Santa Cruz (UCSC) database (33) using splicing-aware STAR aligner (34) for RNA sequencing data. STAR aligner was run in two-pass mode, with known splice junctions supplied in gene transfer format file, obtained from hg38 RefSeq gene annotation table from UCSC database using Table Browser tool (35). The resulting alignments in Binary Alignment Map file format were checked for quality using QualiMap software (36) and Picard tools (37). Picard tools were used to mark PCR/optical duplicate alignments. Custom code was used to filter out contaminating rRNA alignments, using rRNA coordinates for hg38 analysis set reference obtained using UCSC Table Browser tool. The final alignment files were sorted and indexed using Samtools software (38) and visualized using Integrative Genomics Viewer browser (39).

Bioconductor R package RSubread (40) was used to extract raw sequenced fragment counts per transcript using RefSeq hg38 transcript annotation set, as before. Paired-end reads were counted as a single fragment, and multimapping read pairs were counted as a fraction of all equivalent alignments. Raw count data were normalized for library size differences using median ratio method (41), as implemented in DESeq2 R Bioconductor package (42). DESeq2 was also used to perform additional data quality control steps and differential expression analyses. Differentially expressed gene (DEG) expression was visualized as clustered heat maps using Pheatmap R package (43), using log-transformed normalized gene expression values as input. Gene functional and pathway enrichment analyses were performed using R Bioconductor packages clusterProfiler (44) and ReactomePA (45). Additionally, Kyoto Encyclopedia of Genes and Genomes (46) pathways were visualized using Pathview package (47). RNA sequencing data are available in BioProject: https://www.ncbi.nlm.nih.gov/bioproject, accession number PRJNA645253.

To confirm that the sorted cells were pDCs rather than AXL^+^SIGLEC6^+^ DCs (AS-DCs), we analyzed key transcripts associated with each cell type. This confirmed a high expression of the pDC marker TCF4 and a low expression of the AS-DC markers SIGLEC 1, 2, 3, and 6, KLF4, and AXL (Supplemental Table III).

### Statistical analysis

Statistical analyses were carried out with Prism software (GraphPad). Continuous variables were compared using either Student *t* test or ANOVA followed by pairwise Tukey tests. Pearson correlation was used for associations. A *p* value ≤ 0.05 was considered significant. In all figures, error bars indicate SEM unless stated otherwise: *p < 0.05, **p < 0.01, ***p < 0.001, and ****p < 0.0001.

## Results

### Human pDCs produce both IFN-α and TNF-α in response to TLR9 and TLR7 agonists

**FIGURE 1.**
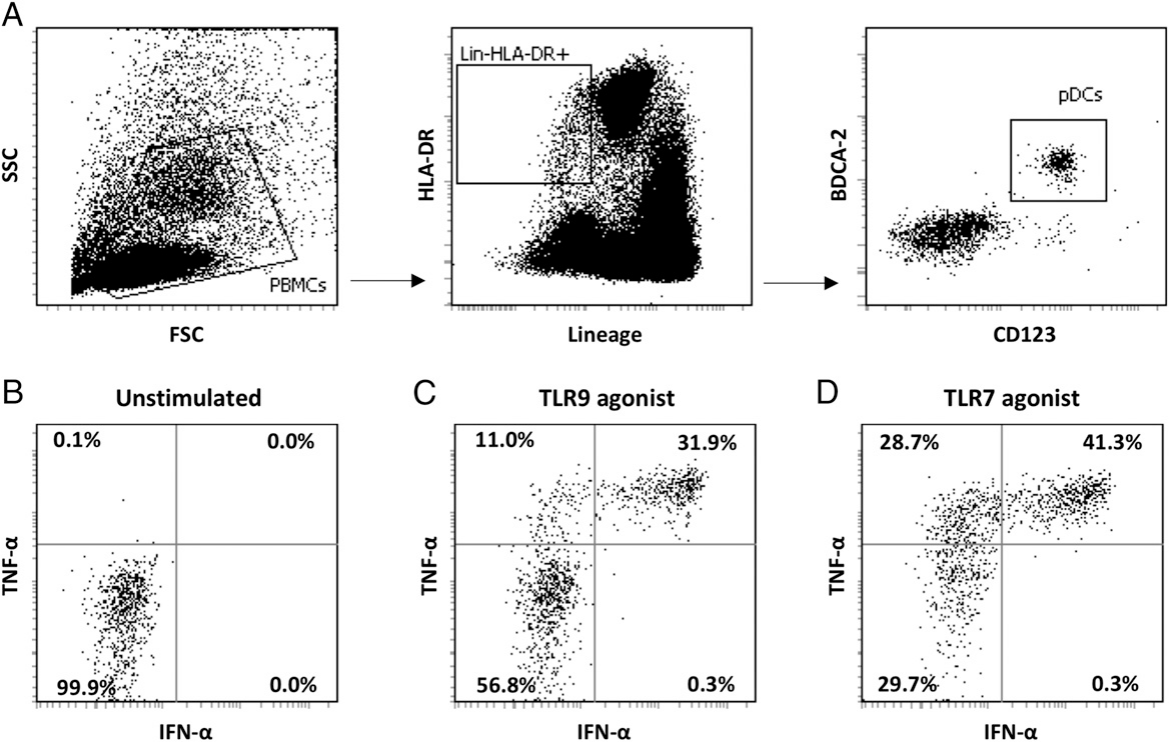
Human pDCs produce both IFN-α and TNF-α in response to TLR9 and TLR7 agonists. (**A**) Gating strategy for human pDCs; pDCs are characterized by the lack of lineage markers (CD3, CD19, CD14, CD56, and CD11c), intermediate to high expression of MHC-II (HLA-DR), and high expression of CD123 and CD303 (BDCA-2). Freshly isolated PBMCs were cultured and stimulated with TLR9 (ODN 2216) or TLR7 (ORN R-2336) agonists for 6 h, and then IFN-α and TNF-α production was detected using intracellular staining. (**B**) Unstimulated pDCs produced no IFN-α and/or TNF-α. (**C** and **D**) Upon stimulation with TLR9 or TLR7 agonists, there were three major pDC populations: 1) nonproducers, 2) TNF-α producers, 3) IFN-α and TNF-α producers. Results shown are representative of three independent experiments.

Although pDCs are mostly recognized for their IFN-α–producing capacity, they are also capable of producing other proinflammatory cytokines. To evaluate this, peripheral blood pDCs were analyzed by flow cytometry for the production of both IFN-α and TNF-α upon stimulation with TLR9 (ODN 2216) or TLR7 (ORN R-2336) agonists. Circulating pDCs produced no IFN-α and/or TNF-α without external stimulation (Fig. 1B). After external stimulation with TLR9 agonist, three major populations of pDCs could be observed: 1) nonproducers, 2) TNF-α producers, and 3) IFN-α and TNF-α producers (Fig. 1C). Similar results could be seen when the cells were also stimulated with TLR7 agonist (Fig. 1D).

### TNF-α regulates IFN-α and TNF-α production in TLR-stimulated pDCs

**FIGURE 2.**
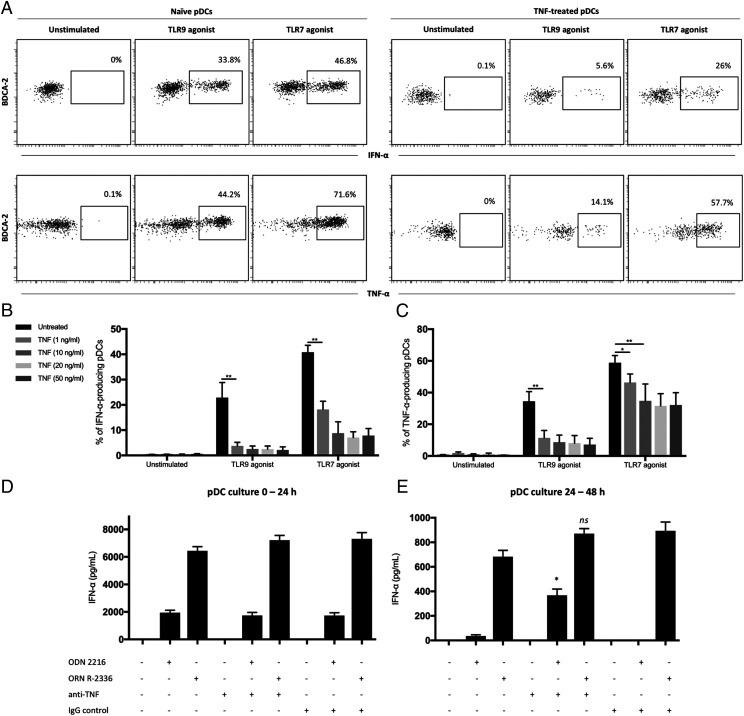
TNF-α regulates IFN-α and TNF-α production in TLR-stimulated pDCs. (**A**) Freshly isolated PBMCs were cultured in the absence or presence of recombinant human TNF-α. After 24 h, PMBCs were washed twice and stimulated with TLR9 (ODN 2216) or TLR7 (ORN R-2336) agonists for 6 h, and then IFN-α and TNF-α production by pDCs was measured using intracellular staining. Results shown are representative of three independent experiments. (**B** and **C**) PBMCs were cultured according to (A) with different concentrations of exogenous TNF-α (0–50 ng/ml). After TLR9 or TLR7 stimulation for 6 h, both IFN-α and TNF-α production by pDCs was measured using intracellular staining. (**D**) Purified pDCs were stimulated with TLR9 or TLR7 agonists in the absence or presence of anti-TNF Ab or isotype control. After 24 h, the supernatants were collected, and IFN-α production was measured by ELISA (0–24 h). (**E**) pDCs were washed twice and restimulated according to (D), and the supernatants were collected after an additional 24 h. IFN-α production was measured by ELISA (24–48 h). Results shown are representative of three independent experiments. Bars represent median value with 95% confidence interval. *p < 0.05, **p < 0.01. *ns*, not significant.

To understand the role of TNF-α on pDC function, we first investigated the effect of TNF-α on cytokine production in the presence or absence of TLR stimulation. Freshly isolated PBMCs were cultured in the absence or presence of different concentrations of recombinant human TNF-α (1–50 ng/ml) for 24 h. We observed no induction of IFN-α and/or TNF-α production in TNF-treated pDCs without exogenous stimulation (Fig. 2A). However, treatment with TNF-α significantly inhibited pDCs’ production of cytokines in response to TLR9 or TLR7 agonists. For TLR9 stimulation, exogenous TNF-α strongly inhibited both IFN-α (Fig. 2B) and TNF-α (Fig. 2C) production by pDCs. For TLR7 stimulation, exogenous TNF-α had a similar effect on inhibiting IFN-α production (Fig. 2B), but a significant reduction in TNF-α production was only seen at higher concentrations (Fig. 2C).

We next investigated whether neutralization of endogenous TNF-α had an impact on IFN-α production. We isolated a pDC-enriched population from PBMCs by negative selection using magnetic beads (purity >92%), and the cells were stimulated with ODN 2216 or ORN R-2336 in the absence or presence of anti-TNF Ab or isotype control. After 24 h, the supernatants were collected, and IFN-α production was measured by ELISA (Fig. 2D). The cells were then washed twice and restimulated with ODN 2216 or ORN R-2336, and the supernatants were collected after an additional 24 h. IFN-α production was measured by ELISA (Fig. 2E). In the first culture (0–24 h), neither anti-TNF–neutralizing Ab nor isotype control altered the levels of IFN-α secreted. However, in the secondary culture (24–48 h), anti-TNF–treated pDCs restimulated with ODN 2216 (TLR9 agonist) could partially maintain IFN-α secretion in comparison with the control-treated pDCs (*p* < 0.05). This effect could not be seen in pDCs restimulated with ORN R-2336 (TLR7 agonist) as the levels of IFN-α secreted were similar in both anti-TNF– and control-treated pDCs.

### RNA sequencing data generation

We sought to investigate how TNF-α regulates TLR-mediated cytokine production and induces further transcriptional changes in human pDCs. pDCs from healthy donors (*n* = 3) were purified by negative selection using magnetic beads. The pre-enriched pDCs (purity >85%) from each donor were divided into two aliquots before they were cultured in the presence or absence of human recombinant TNF-α (10 μl/ml) for 18 h. After incubation, untreated and TNF-treated pre-enriched pDCs from all three donors (*n* = 6) were washed and sorted based on CD304 (BDCA-4) expression to achieve purity >99%.

Principal component analysis demonstrated that the main source of variation in each sample derived from the treatment with TNF-α (Supplemental Fig. 1A). In total, the analysis indicated 1800 DEGs at <5% false discovery rate between untreated and TNF-treated pDCs. The top 100 upregulated and downregulated genes by TNF-α in pDCs can be found in Supplemental Tables I and II, respectively.

### TNF-α promotes transcriptional changes associated with Ag processing and presentation

**FIGURE 3.**
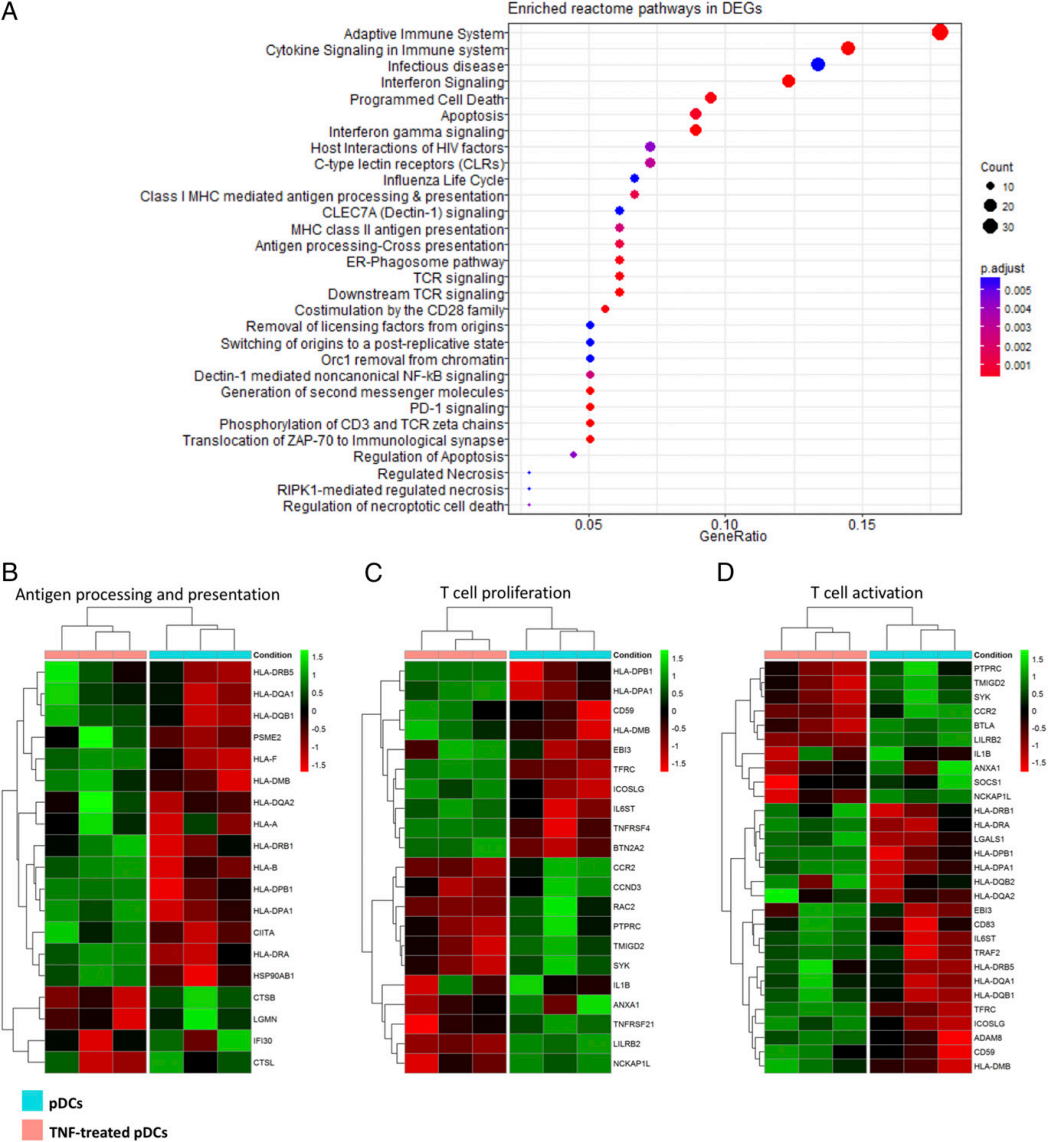
TNF-α promotes transcriptional changes associated with Ag processing and presentation. (**A**) Enriched Reactome pathways in DEGs upregulated by TNF-α in pDCs compared with untreated pDCs. (**B**–**D**) Heat maps showing that TNF-α promotes *DEGs associated with Ag processing and presentation, T cell proliferation, and T cell activation pathways in pDCs.

**FIGURE 4.**
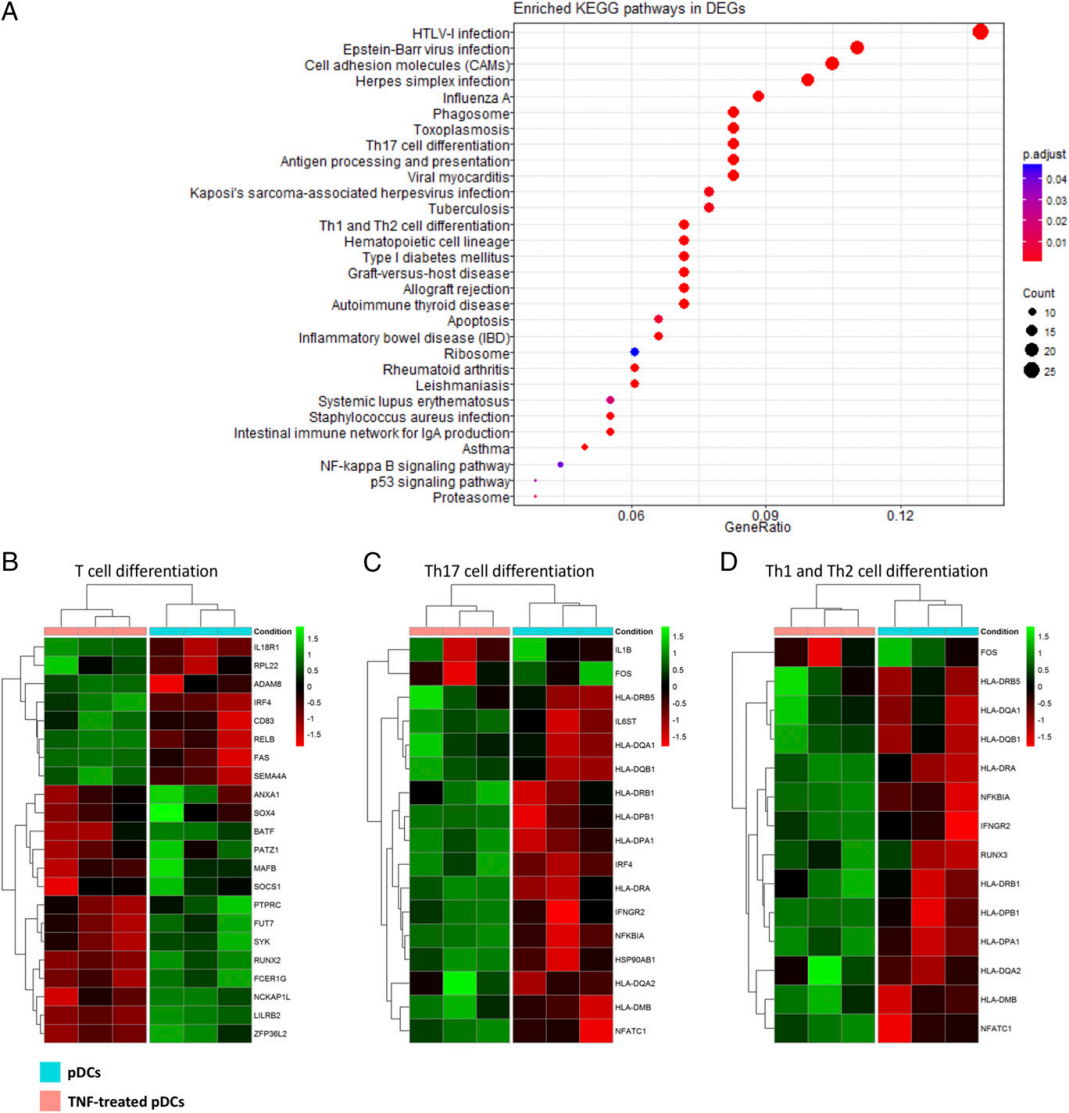
TNF-α promotes transcriptional changes associated with T cell differentiation. (**A**) Enriched KEGG pathways in DEGs upregulated by TNF-α in pDCs. (**B**–**D**) Heat maps showing that TNF-α promotes DEGs associated with T cell differentiation toward Th17, Th1, and Th2 pathways in pDCs.

TNF-α induced the upregulation of genes in pDCs, which were particularly enriched for pathways associated with MHC-II Ag processing and presentation, Th17 differentiation, Th1 and Th2 differentiation, MHC class I Ag processing and cross-presentation, induction of TCR signaling and costimulation of CD28, phosphorylation of CD3 and TCR ζ-chains, and translocation of ZAP70 to immunological synapse among other pathways. A detailed presentation of enriched Reactome and Kyoto Encyclopedia of Genes and Genomes pathways in DEGs can be seen in Figs. 3A and 4A, respectively as well as Supplemental Fig. 1B. Among the most enriched biological processes induced by TNF-α were found to be lymphocyte aggregation, T cell activation, immune response–activating cell surface receptor signaling, Ag processing and presentation of exogenous Ag, and T cell costimulation. Regarding the enriched cellular components in DEGs, these included units and functions mainly related to Ag processing and presentation (Supplemental Fig. 2A); for instance, endocytic vesicle membrane, MHC-II protein complex, clathrin-coated vesicle membrane, and others.

Among the upregulated genes in TNF-treated pDCs compared with untreated pDCs were HLAs corresponding to MHC class I (*HLA-A*, *HLA-B*, and *HLA-F*) as well as MHC-II (*HLA-DPA1*, *HLA-DPB1*, *HLAD-DRA*, *HLA-DRB1*, *HLA-DRB5*, *HLA-DQA1*, *HLA-DQA2*, *HLA-DQB1*, and *HLA-DMB*).

Analyzing the RNA sequencing data further for the biological effects of TNF-α on the function of pDCs, DEGs associated with positive regulation of T cell proliferation and activation were particularly enriched (Fig. 3B). Upregulated genes included costimulatory molecules (*CD80*, *CD86*, *CD83*), molecules promoting endocytosis (*CD59*) and cell adhesion (*ADAM8*), ICOS ligand (*ICOSLG*), and IL-27 subunit β (*EBI3*). In contrast, downregulated genes included *CCR2*, *PTPRC*, *SYK*, *IL1B*, *LILRB2*, and *SOCS1*, among others. TNF-α also upregulated genes associated with T cell differentiation (*RPL22*, *ADAM8*, *IRF4*, *CD83*, *RELB*, *FAS*, and *SEMA4A*), particularly toward Th17, Th1, and Th2 pathways (Fig. 4B).

### TNF-α inhibits the transcription of genes encoding members of the TLR signaling cascade

**FIGURE 5.**
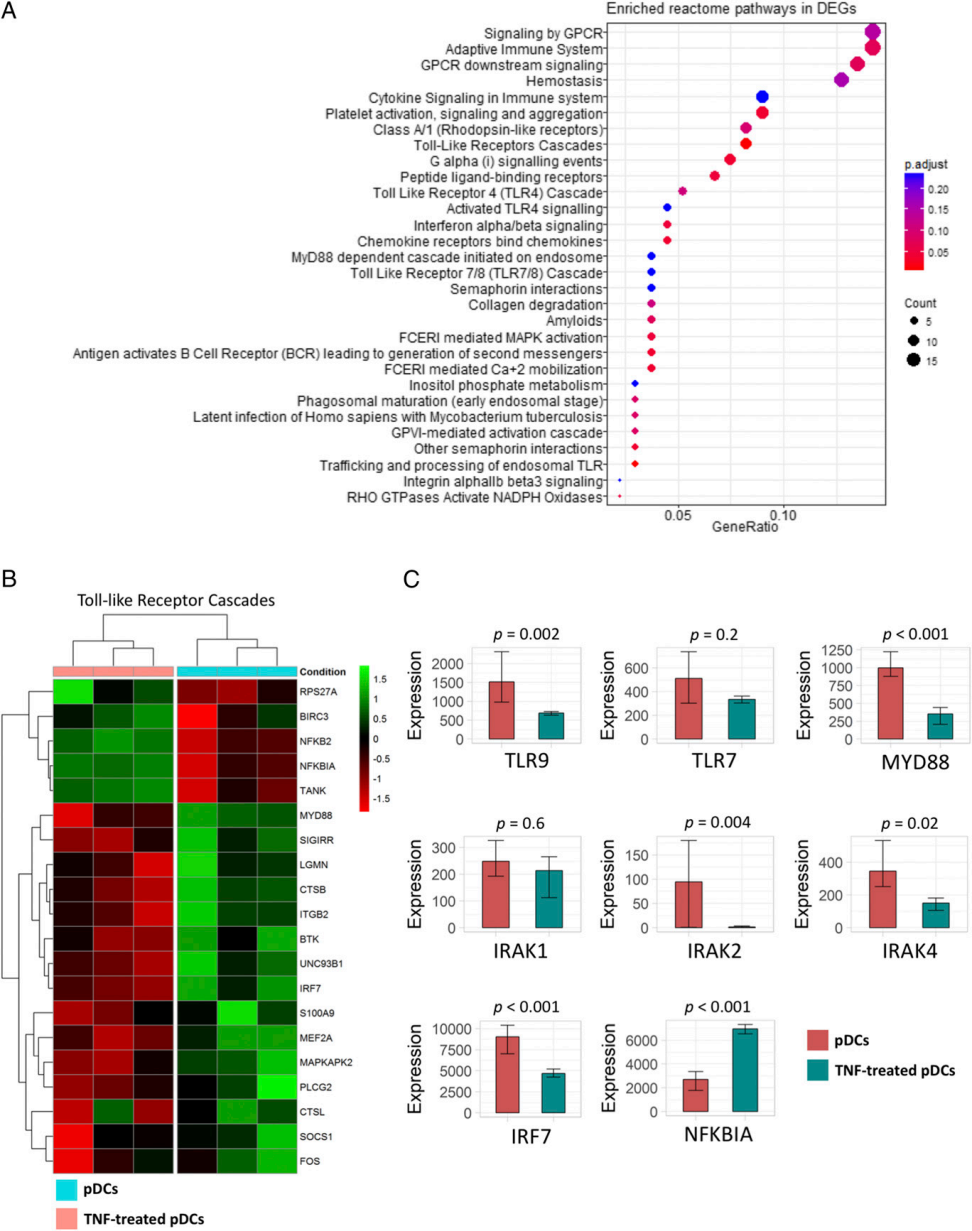
TNF-α inhibits TLR cascade signaling pathways. (**A**) Enriched Reactome pathways in DEGs downregulated by TNF-α in pDCs. (**B**) Heat maps showing that TNF-α promotes DEGs associated with negative regulation of TLR cascade signaling pathway in pDCs. (**C**) DEGs in TNF-treated versus untreated pDCs associated with negative regulation of TLR-mediated type I IFN production.

Despite the transcriptional changes toward Ag processing and presentation as well as T cell activation and differentiation, TNF-α seemed to negatively regulate other functions of pDCs. Among the downregulated genes in TNF-treated pDCs compared with untreated pDCs, there were enriched pathways associated with G protein–coupled receptor cascade signaling, TLR cascade signaling and IFN-α/β secretion (MyD88 and MAPK signaling pathways), phagosomal maturation (early endosomal stage), and regulation of trafficking and processing of endosomal TLRs (Fig. 5A, Supplemental Fig. 2B).

As type I IFN and proinflammatory cytokine production by pDCs is primarily mediated by TLR7 and TLR9 ligation with nucleic acids in early endosomes, the effect of TNF-α in TLR cascade signaling was interrogated in the RNA sequencing data analysis. Not surprisingly, since the in vitro experiments confirmed that TNF-α inhibits the secretory function of pDCs, there was a significant downregulation of genes encoding intracellular proteins and kinases mediating the phosphorylation of IRF7, NF-κB, and AP-1 with the eventual outcome of the production of type I IFNs and proinflammatory cytokines (Fig. 5B). There was a statistically significant reduction in the expression levels of *TLR9*, *MyD88*, *IRAK2*, *IRAK4*, and *IRF7*. In contrast, there was upregulation of the NF-κB inhibitor (*NFKBIA*) known to block the translocation of NF-κB to the nucleus (Fig. 5C). In addition, TNF-treated pDCs presented a significant downregulation of common IFN-stimulated genes, including *SOCS*, *IFI30*, *IRF7*, *IFITM2*, *IFITM3*, and *OAS1*, whereas there was upregulation of other IFN-stimulated genes such as *BST2*, *IRF4*, *NUP62*, and *IFNGR2* (Supplemental Fig. 2C).

### TNF-α upregulates maturation markers and costimulatory molecules on pDCs

**FIGURE 6.**
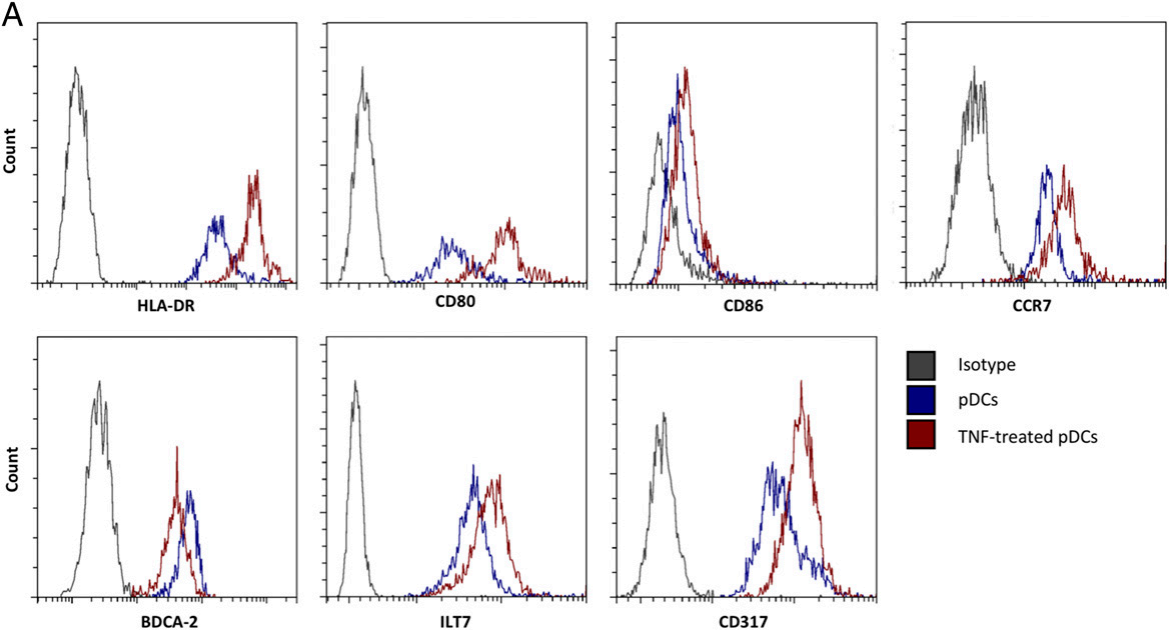
TNF-α upregulates costimulatory molecules and maturation markers on pDCs. pDCs were purified from freshly isolated PBMCs and cultured in the absence or presence of recombinant human TNF-α. After 24 h, pDCs were analyzed by flow cytometry. Fluorescence intensity is shown on the *x*-axis. Results shown are representative of three independent experiments. (**A**) TNF-α upregulates HLA-DR (MHC-II), costimulatory molecules such as CD80 and CD86, and CCR7 but downregulates pDC-specific markers such as BDCA-2 (CD303). TNF-α also upregulates receptors related to type I IFN regulation such as ILT7 (CD85g) and CD317 (BST2; tetherin). Results shown are representative of three independent experiments.

To validate the RNA sequencing data at protein level, purified pDCs were cultured in the presence or absence of exogenous TNF-α for 24 h before the expression of surface molecules was then measured by flow cytometry (Fig. 6). TNF-α strongly upregulated maturation markers such as HLA-DR (MHC-II) and CCR7 (CD197) as well as the costimulatory molecules CD80 and CD86 on pDCs. Molecules related to the negative regulation of IFN-α such as ILT7 (CD85j) and CD317 (BST2, tetherin) were also upregulated. In contrast, TNF-α downregulated pDC-specific markers such as BDCA-2 (CD303).

### TNF-α enhances Ag uptake and processing by pDCs

**FIGURE 7.**
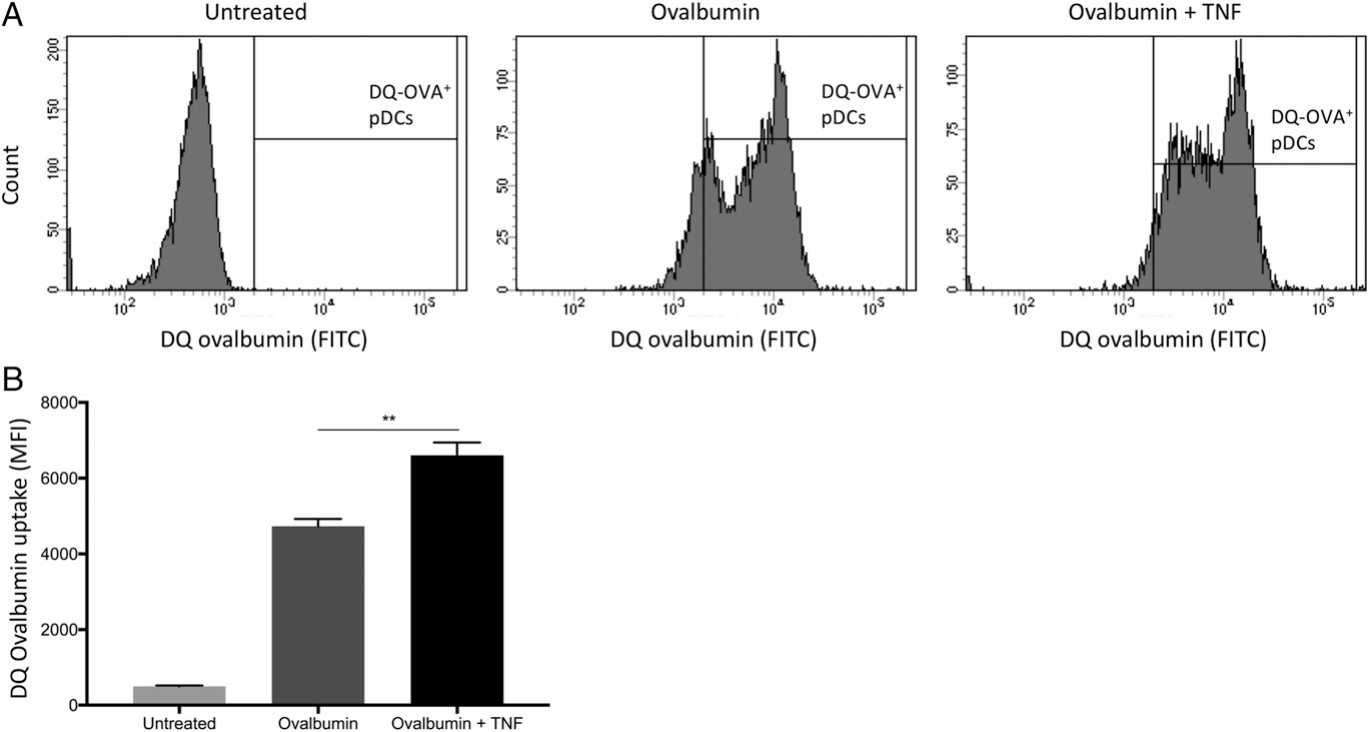
TNF-α enhances Ag uptake and processing by pDCs. (**A**) pDCs were purified by freshly isolated PBMCs and cultured alone or with 10 μg/ml DQ OVA in the presence or absence of 50 ng/ml TNF-α for 18 h. Ag uptake and processing was measured by flow cytometry based on mean fluorescence intensity (MFI). One representative experiment is shown out of three independent experiments. (**B**) Summary of the DQ OVA uptake of untreated pDCs, DQ OVA–treated pDCs, and DQ OVA/TNF–treated pDCs. Data are presented as means ± SEM for three independent experiments. **p < 0.005.

Based on RNA sequencing data analysis, we further cultured pDCs to assess whether TNF-α could enhance Ag uptake and processing. First, pDCs were purified from freshly isolated PBMCs as described above and were then cultured alone or with 10 μg/ml DQ OVA in the presence or absence of 50 ng/ml TNF-α. After 18 h, pDCs were harvested, and DQ OVA processing was analyzed by flow cytometry based on the level of mean fluorescence intensity. Almost all pDCs in the presence or absence of TNF-α were able to internalize and process DQ OVA as compared with the negative control (Fig. 7A). Interestingly, TNF-α significantly enhanced the uptake of DQ OVA by pDCs demonstrating an increased Ag processing capacity (Fig. 7B). These results confirmed that TNF-α favored Ag uptake and processing by human pDCs.

### TNF-α–treated pDCs enhance T cell proliferation and activation

**FIGURE 8.**
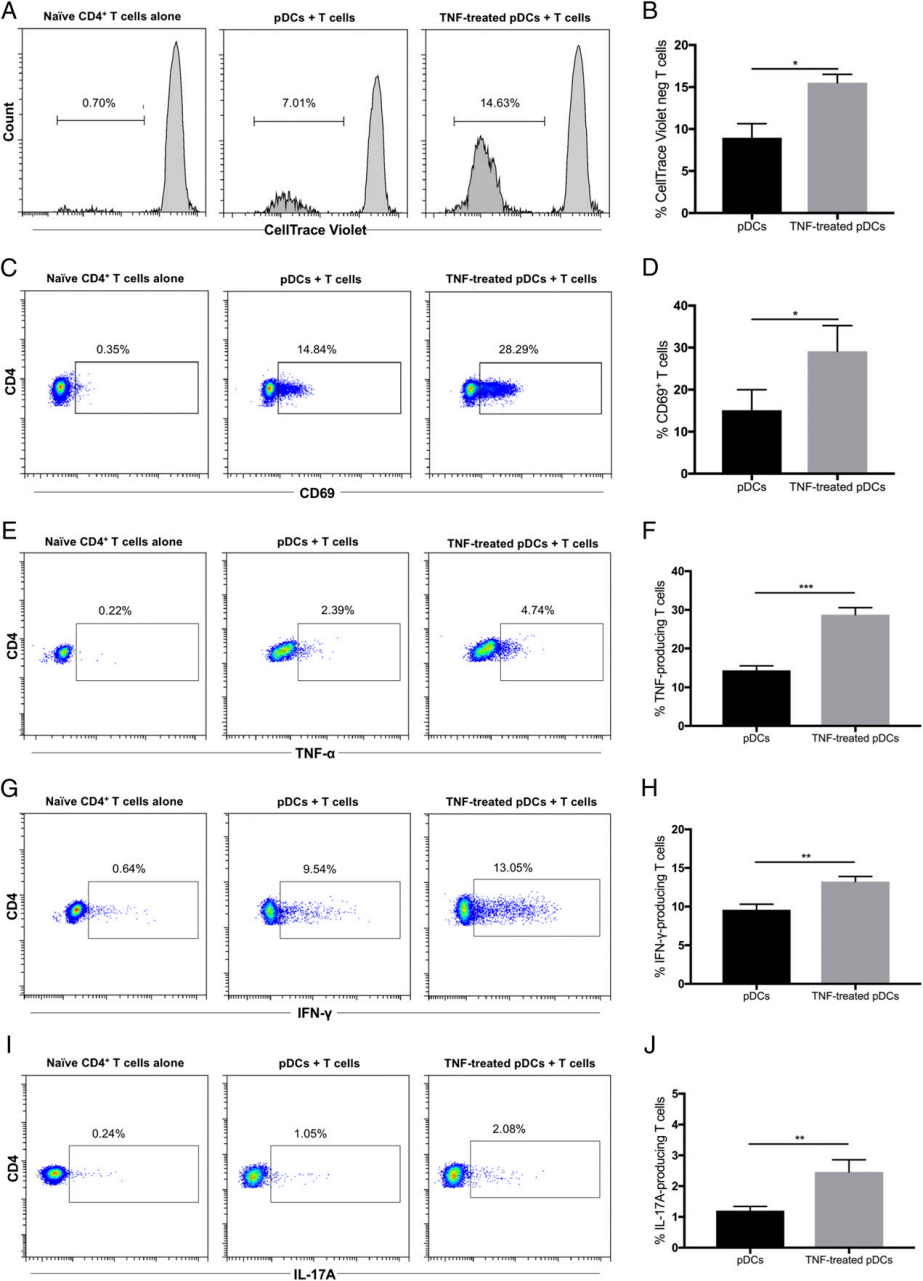
TNF-α–treated pDCs enhance T cell proliferation and activation. (**A**) Allogeneic naive CD4^+^ T cells were labeled with CellTrace Violet and cultured alone or with pDCs or TNF-α–treated pDCs for 5 d. T cell proliferation was analyzed by flow cytometry based on CellTrace Violet dilution. One representative experiment is shown out of three independent experiments. (**B**) Average percentage of proliferated CD4^+^ T cells cocultured with pDCs or TNF-α–treated pDCs (*n* = 3). (**C**) Expression of CD69 on CD4^+^ T cells from the cultures shown in (A). One representative experiment is shown out of three independent experiments. (**D**) Average expression of CD69 on CD4^+^ T cells cocultured with pDCs or TNF-α–treated pDCs (*n* = 3). (**E**–**J**) Allogeneic naive CD4^+^ T cells were cultured alone or with pDCs or TNF-α–treated pDCs for 5 d. Percentage of TNF-α (E), IFN-γ (G), and IL-17A (I) production by CD4^+^ T cells was measured by intracellular staining. One representative experiment is shown out of three independent experiments. Average percentage of TNF-α (F), IFN-γ (H), and IL-17A (J) produced CD4^+^ T cells cocultured with pDCs or TNF-α–treated pDCs (*n* = 3). *p < 0.05, **p < 0.001, ***p < 0.001.

Following RNA sequencing data analysis, we performed a series of in vitro allogeneic pDC–naive CD4^+^ T cell cocultures to evaluate whether TNF-α could enhance the ability of pDCs to induce T cell activation. First, pDCs were cultured in the presence or absence of exogenous TNF-α (10 ng/ml) for 24 h. After washing thoroughly, pDCs were cocultured with allogeneic naive CD4^+^ T cells for 5 d. T cell proliferation was assessed based on CellTrace Violet dilution upon cell division using flow cytometry. Both sets of pDCs could induce T cell proliferation without exogenous stimulation (Fig. 8A). However, TNF-treated pDCs were more efficient as they induced a significantly higher percentage of proliferating T cells (Fig. 8B). Similarly, TNF-treated pDCs induced a higher expression of CD69 on CD4^+^ T cells (Fig. 8C, 8D). Regarding cytokine production, TNF-treated pDCs induced a notably higher production of TNF-α (Fig. 8E, 8F, 2.39 versus 4.74%) and IFN-γ (Fig. 8G, 8H, 9.54 versus 13.05%) as well as IL-17A (Fig. 8I, 8J, 1.05 versus 2.08%) in comparison with untreated pDCs. Collectively, these results confirmed that TNF-α enhances the capacity of pDCs to induce T cell proliferation and activation favoring the production of Th1 and Th17 cytokines.

## Discussion

Apart from the major role of pDCs as type I IFN–producing cells, pDCs are able to capture, process, and present Ags to CD4^+^ T cells. However, the regulation of these functions was previously elusive. Previous studies reported the cross-regulation of TNF-α and IFN-α during in vitro stimulation of pDCs with influenza virus; however, the precise mechanism for this effect was not fully addressed (31). It was also shown that TNF-α in combination with IFN-α promotes induction of CD80 and CD86 on pDCs (3). In this study, we demonstrated that TNF-α not only inhibited IFN-α production even at minimal concentrations but also had a similar regulatory effect on autologous TNF-α production, predominantly in a TLR9-mediated manner.

Although we have elucidated the regulation of the well-known pDC, it is important to consider other related DC subsets more recently described. The traditional pDC population is characterized by the expression of genes associated with pathogen sensing and induction of type I IFNs as well as the master regulator transcription factor *TCF4* (48). A novel DC subset was previously described with a unique gene signature (*AXL*, *SIGLEC6*, *SIGLEC1*, and *SIGLEC2*) sharing features of both cDCs and pDCs (“AS-DCs”) (49, 50). AS-DCs are characterized by the expression of HLA-DR, CD34, and BDCA-3 but, unlike pDCs, they demonstrate CD11c expression as cDCs (50, 51). In contrast, both the expression of CD123 and BDCA-2 appears to be lower. In our data, RNA sequencing analysis of sorted pDCs showed a high expression of the main transcription factor defining pDC lineage TCF4 (E2-2) but no expression of the AS-DC markers SIGLEC-6 (CD33L), SIGLEC-1 (CD169), and SIGLEC-2 (CD22) and very minimal expression of AXL and SIGLEC-3 (CD33). In addition, TNF-α did not upregulate any of the AS-DC markers but promoted Ag-presenting properties by different transcriptomic changes.

Another single-cell RNA sequencing study revealed the diversification of human pDCs in response to influenza virus into three phenotypes (P1-, P2-, and P3-pDCs) with distinct transcriptional profiles and functions (9). The P3-pDCs were identified by higher expression of CD80, CD86, other costimulatory molecules, and CCR7; they also had a high potency of activating T cells mainly toward Th2 responses. We found that TNF-α can induce the upregulation of all costimulatory molecules on pDCs alongside CCR7, favoring the activation of T cells mainly toward Th1 and Th17 responses. Another novel pDC subset characterized as CD2^hi^CD5^+^CD81^+^ was also shown to induce strong T and B cell activation but not to be able to secrete type I IFNs (52). In our study, RNA sequencing data of TNF-treated pDCs revealed a downregulation of this pDC-specific gene signature associated with pathogen sensing (*IRF7*, *TLR9*, *SLC15A4*, and *PACSIN1*) and secretion (*DERL3*, *LAMP5*, and *SCAMP5*) as well as *TCF4* alongside its binding targets (*SLA2*, *PTCRA*, and *PTPRCAP*). Hence, TNF-α strongly influenced the transcriptional profile of pDCs by downregulating their classical pathways and upregulating genes (e.g., *LY86*) mostly related to the cDC phenotype. Our data suggest that TNF-α can alter intracellular pathways and may interfere with phosphorylation of transcription factors such as IRF7 and IκB involved in cytokine production and other known functions of pDCs.

Other cytokines such as IL-21 have been shown to have a regulatory impact on the function of pDCs promoting the secretion granzyme B, which impairs their capacity to induce T cell proliferation (53). In contrast, we showed that TNF-α enhanced the immunogenic properties of pDCs toward Ag processing and presentation and T cell activation and proliferation. Regarding TNFR superfamily, TNF-α downregulated *TNFRSF1A* (TNFR 1A; CD120a) but not *TNFRSF1B* (TNFR 1B; CD120b) and *TNFRSF6B* (decoy receptor 3; TR6; M68), whereas it upregulated *TNFRSF4* (OX40; CD134), *FAS* (Fas receptor; Apo-1; CD95), and *CD40* (Bp50; CD40). Interestingly, TNF-α promoted the downregulation of *IFNAR1* and *IFNAR2* and the upregulation of *IFNGR1* and *IFNGR2*, receptors for IFN-γ. This may suggest that changes in TNFR1 and TNFR2 receptor expression accompany the functional changes in pDCs that we observed. Although TNFR1 can mediate apoptosis, such an effect did not appear consistent with our results, with an increase in T cell activation by pDCs after TNF exposure. Changes in receptor expression at a protein level and studying their functional significance require further work. In future work, we will also study what signaling mechanisms explain the differential responses to TLR7 and TLR9 ligands.

Linking these data to human disease, anti-TNF treatment has been associated with lupus-like symptoms as well as an induction of IFN signature in peripheral blood (30, 31, 54, 55). The synovium of rheumatoid arthritis patients was reported to contain pDCs able to activate T cells more efficiently (56). TNF-α as one of the main pathogenic cytokines driving synovial inflammation may alter local pDC function enhancing Ag presentation and promoting Th1 and Th17 cell differentiation. In addition, anti-TNF treatment in patients with paradoxical psoriasis prolonged type I IFN production by pDCs through inhibition of their maturation (57).

In summary, our data elucidate the plasticity of pDC function according to their inflammatory environment. Although pDCs possess weak Ag-presenting properties, TNF-α can enhance these functions by switching their main role as IFN-α–producing cells to a more cDC phenotype. This cytokine therefore plays a key role in modulating the functional status of pDCs and may regulate IFN-α–mediated aspects of a range of autoimmune and inflammatory diseases.

## Supplementary Material

Data Supplement
