# Supplementary material for: TNF-α Regulates Human Plasmacytoid Dendritic Cells by Suppressing IFN-α Production and Enhancing T Cell Activation
Source: J Immunol. 2021 Jan 13;206(4):785–96. doi: 10.4049/jimmunol.1901358 (PMC7851743; doi:10.4049/jimmunol.1901358)
Supplement: Data Supplement [file JI_1901358.zip › JI_1901358_Supplemental_Material_1.pdf]

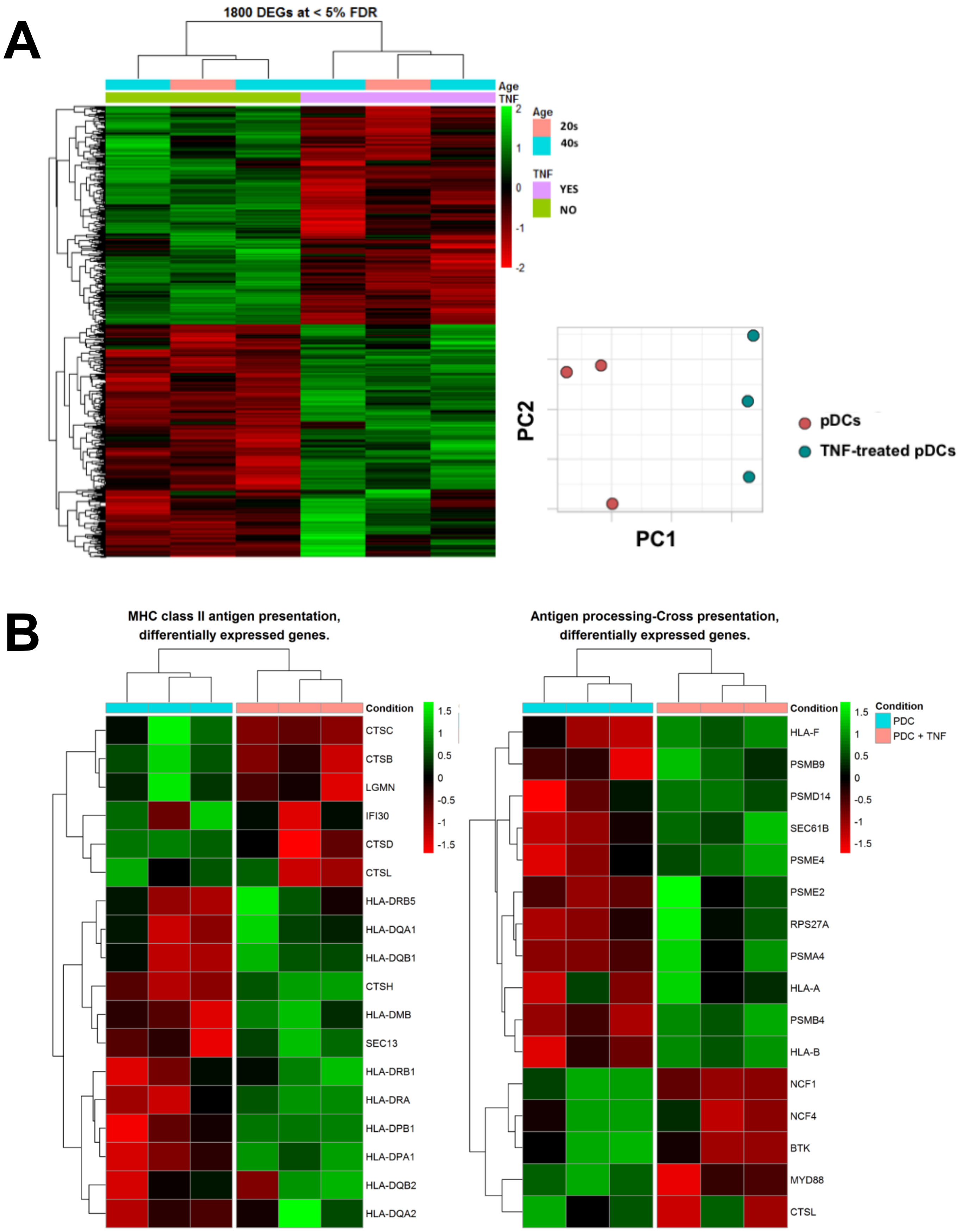

Supplemental Figure 1.

A: All 1,800 differentially expressed genes (DEGs) at < 5% false discovery rate (FDR) between untreated and TNF-treated pDCs. Principal component analysis (PCA) shows that the main source of variation in the data derives from the treatment with TNF-α.

B: Heatmaps showing the differentially expressed genes (DEGs) in TNF-treated vs. untreated pDCs related to MHC class II antigen presentation and antigen processing/cross presentation pathways.

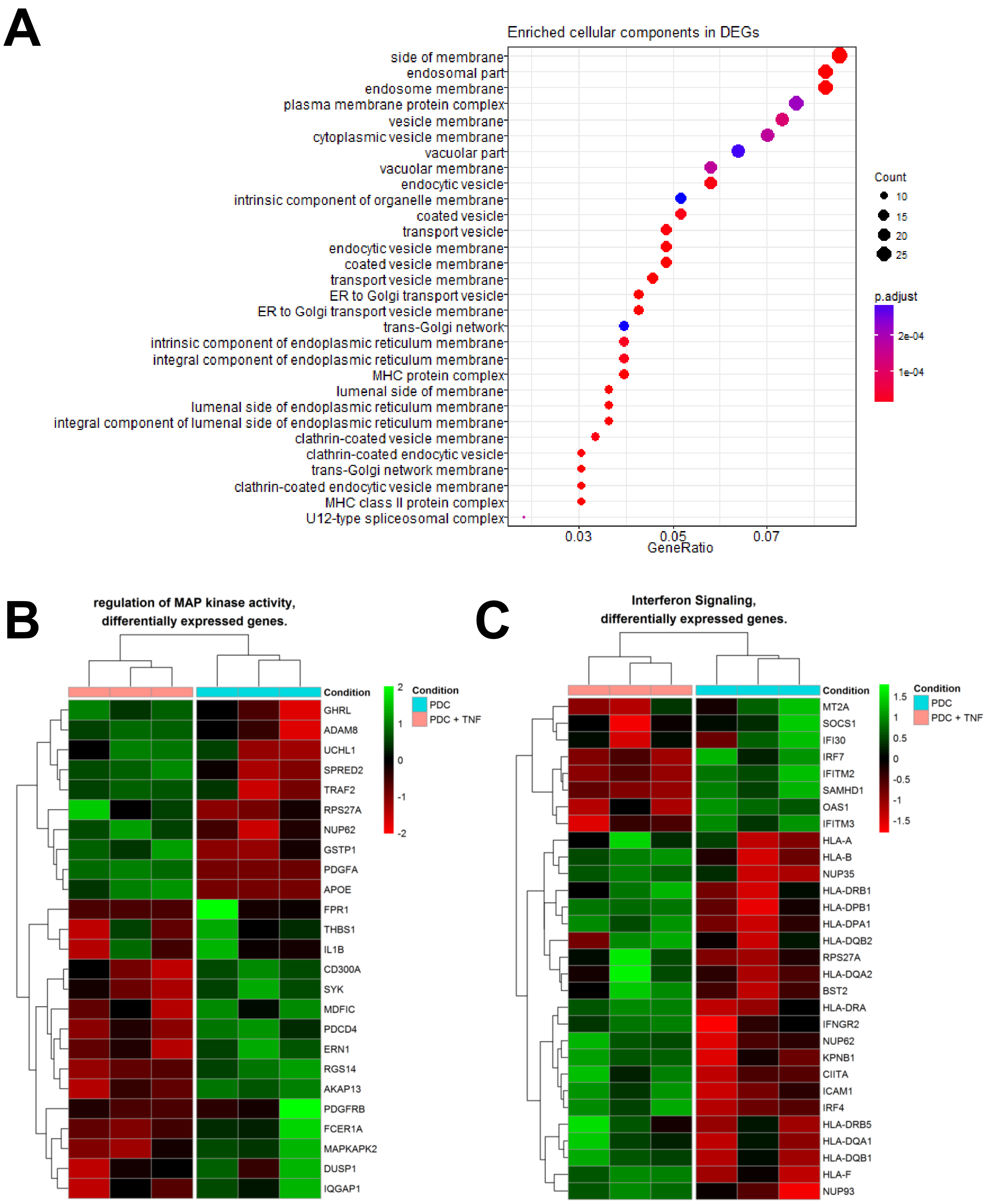

Supplemental Figure 2.

A: Enriched cellular components in differentially expressed genes (DEGs) upregulated by TNF-α in pDCs
B: Heatmap showing the differentially expressed genes (DEGs) in TNF-treated vs. untreated pDCs related to negative regulation of MAPK kinase activity.
C: Heatmap of differentially expressed genes in TNF-treated vs. untreated associated with IFN signaling pathway.

Supplemental Table 1. Top 100 genes upregulated by TNF-α in pDCs.

pDCs vs. TNF-treated pDCs							
**Gene**	**Fold Change (log2)**	***P* value**	**FDR**	**Gene**	**Fold Change (log2)**	***P* value**	**FDR**
CRIP2	6.846	< 0.001	< 0.001	HIVEP3	3.31	< 0.001	< 0.001
FXYD2	3.599	< 0.001	< 0.001	SWAP70	2.38	< 0.001	< 0.001
FXYD6-FXYD2	3.575	< 0.001	< 0.001	HLA-DRB1	1.18	< 0.001	< 0.001
ANO9	4.52	< 0.001	< 0.001	MGLL	1.619	< 0.001	< 0.001
CRIP1	2.803	< 0.001	< 0.001	SYNGR2	1.27	< 0.001	< 0.001
TYW3	2.74	< 0.001	< 0.001	FSCN1	2.109	< 0.001	< 0.001
TNFRSF4	3.115	< 0.001	< 0.001	NME1	2.258	< 0.001	< 0.001
HDGFRP3	4.35	< 0.001	< 0.001	TVP23A	2.441	< 0.001	< 0.001
HLA-DQA1	2.169	< 0.001	< 0.001	BLVRB	2.908	< 0.001	< 0.001
CCDC28B	2.696	< 0.001	< 0.001	SEC61B	1.157	< 0.001	< 0.001
BCL2A1	2.589	< 0.001	< 0.001	TMEM120B	1.773	< 0.001	< 0.001
TMEM138	2.036	< 0.001	< 0.001	GPATCH4	2.068	< 0.001	< 0.001
TFEC	2.382	< 0.001	< 0.001	NCCRP1	3.587	< 0.001	< 0.001
UNQ6494	5.108	< 0.001	< 0.001	RHOF	1.689	< 0.001	< 0.001
CTSH	2.375	< 0.001	< 0.001	DGAT2	3.027	< 0.001	< 0.001
KDM2B	2.617	< 0.001	< 0.001	TXN	1.243	< 0.001	< 0.001
PDGFA	4.535	< 0.001	< 0.001	DDB2	3.466	< 0.001	< 0.001
CDKN1A	2.015	< 0.001	< 0.001	NFKBIA	1.352	< 0.001	< 0.001
HLA-DQB1	1.814	< 0.001	< 0.001	DCANP1	2.159	< 0.001	< 0.001
NEK6	2.364	< 0.001	< 0.001	DNPH1	1.714	< 0.001	< 0.001
CD83	1.929	< 0.001	< 0.001	TIGIT	3.193	< 0.001	< 0.001
EDNRB	4.598	< 0.001	< 0.001	ICAM1	1.579	< 0.001	< 0.001
FAS	3.784	< 0.001	< 0.001	GRHPR	1.572	< 0.001	< 0.001
LAGE3	2.247	< 0.001	< 0.001	HLA-F	1.592	< 0.001	< 0.001
RELB	2.367	< 0.001	< 0.001	TLCD1	2.062	< 0.001	< 0.001
CRYZ	1.981	< 0.001	< 0.001	FDPS	1.324	< 0.001	< 0.001
IL6ST	2.175	< 0.001	< 0.001	CYB5A	1.984	< 0.001	< 0.001
GPX4	1.646	< 0.001	< 0.001	CFLAR	1.43	< 0.001	< 0.001
LSR	2.904	< 0.001	< 0.001	PPIL1	3.01	< 0.001	< 0.001
GGT1	3.31	< 0.001	< 0.001	TTC39A	2.011	< 0.001	< 0.001
KREMEN2	3.689	< 0.001	< 0.001	PPA1	2.043	< 0.001	< 0.001
BID	1.578	< 0.001	< 0.001	RPSA	1.146	< 0.001	< 0.001
MAD2L2	1.817	< 0.001	< 0.001	LRRC75A-AS1	1.299	< 0.001	< 0.001
UQCC2	2.382	< 0.001	< 0.001	BIRC3	1.575	< 0.001	< 0.001
DPCD	3.829	< 0.001	< 0.001	TCEB3	1.299	< 0.001	< 0.001
SEMA4A	1.986	< 0.001	< 0.001	HLA-DPB1	1.245	< 0.001	< 0.001
DNASE1L3	1.411	< 0.001	< 0.001	RSL1D1	1.27	< 0.001	< 0.001
HSD17B10	1.858	< 0.001	< 0.001	ADAT2	2.994	< 0.001	< 0.001
BTN2A2	2.168	< 0.001	< 0.001	SMS	2.337	< 0.001	< 0.001
TFRC	1.855	< 0.001	< 0.001	FEZ1	3.242	< 0.001	< 0.001
SSH1	2.579	< 0.001	< 0.001	ATOX1	1.333	< 0.001	< 0.001
CX3CR1	2.33	< 0.001	< 0.001	LINC01268	3.205	< 0.001	< 0.001
WNT10A	2.311	< 0.001	< 0.001	MRPL14	1.615	< 0.001	< 0.001
ANK3	3.667	< 0.001	< 0.001	HLA-DRA	1.116	< 0.001	< 0.001
MARCKS	2.336	< 0.001	< 0.001	IL10RA	1.289	< 0.001	< 0.001
CD59	2.135	< 0.001	< 0.001	HLA-DRB5	1.042	< 0.001	< 0.001
TIMM10	1.838	< 0.001	< 0.001	CLIC2	1.878	< 0.001	< 0.001
TNFAIP2	2.723	< 0.001	< 0.001	SPRED2	2.875	< 0.001	< 0.001
HLA-DQB2	1.817	< 0.001	< 0.001	GRPEL1	1.667	< 0.001	< 0.001
HLA-DQA2	1.857	< 0.001	< 0.001	NKG7	2.605	< 0.001	< 0.001

Supplemental Table 2. Top 100 genes downregulated by TNF-α in pDCs.

pDCs vs. TNF-treated pDCs							
**Gene**	**Fold Change (log2)**	***P* value**	**FDR**	**Gene**	**Fold Change (log2)**	***P* value**	**FDR**
CST3	-2.557	< 0.001	< 0.001	IGFBP3	-2.742	< 0.001	< 0.001
HS3ST1	-4.792	< 0.001	< 0.001	NCF1B	-1.548	< 0.001	< 0.001
S100A4	-2.993	< 0.001	< 0.001	RUNX2	-1.786	< 0.001	< 0.001
RASD1	-3.091	< 0.001	< 0.001	SYNGR1	-2.075	< 0.001	< 0.001
S100A6	-2.513	< 0.001	< 0.001	RN7SL1	-1.195	< 0.001	< 0.001
LOC100507600	-3.04	< 0.001	< 0.001	GAPT	-1.333	< 0.001	< 0.001
BTLA	-4.467	< 0.001	< 0.001	CXCL16	-2.246	< 0.001	< 0.001
SCN9A	-2.466	< 0.001	< 0.001	LOC101928034	-2.745	< 0.001	< 0.001
PLXNA4	-3.152	< 0.001	< 0.001	LILRB2	-3.286	< 0.001	< 0.001
IFITM2	-2.324	< 0.001	< 0.001	RNF166	-2.115	< 0.001	< 0.001
ACY3	-2.246	< 0.001	< 0.001	RAB11FIP4	-3.369	< 0.001	< 0.001
PLD4	-1.91	< 0.001	< 0.001	GAS6	-1.888	< 0.001	< 0.001
SPNS3	-2.95	< 0.001	< 0.001	CMTM7	-2.49	< 0.001	< 0.001
PLP2	-1.707	< 0.001	< 0.001	MAPKAPK2	-2.06	< 0.001	< 0.001
LIME1	-2.156	< 0.001	< 0.001	LILRA4	-1.351	< 0.001	< 0.001
MS4A6A	-1.85	< 0.001	< 0.001	EPS8L2	-2.669	< 0.001	< 0.001
TXNIP	-1.819	< 0.001	< 0.001	FCER1G	-1.302	< 0.001	< 0.001
METTL7A	-2.991	< 0.001	< 0.001	C1orf162	-1.706	< 0.001	< 0.001
RNA5S1	-1.692	< 0.001	< 0.001	TNFRSF17	-2.301	< 0.001	< 0.001
RNA5S10	-1.692	< 0.001	< 0.001	HHEX	-1.698	< 0.001	< 0.001
RNA5S11	-1.692	< 0.001	< 0.001	LINGO3	-3.276	< 0.001	< 0.001
RNA5S12	-1.692	< 0.001	< 0.001	HPCAL1	-2.144	< 0.001	< 0.001
RNA5S13	-1.692	< 0.001	< 0.001	CD164	-1.315	< 0.001	< 0.001
RNA5S14	-1.692	< 0.001	< 0.001	RN7SL2	-1.166	< 0.001	< 0.001
RNA5S15	-1.692	< 0.001	< 0.001	UCP2	-1.378	< 0.001	< 0.001
RNA5S16	-1.692	< 0.001	< 0.001	RGS14	-2.451	< 0.001	< 0.001
RNA5S17	-1.692	< 0.001	< 0.001	NCF1C	-1.264	< 0.001	< 0.001
RNA5S2	-1.692	< 0.001	< 0.001	LRRC25	-3.003	< 0.001	< 0.001
RNA5S3	-1.692	< 0.001	< 0.001	LOC102724297	-2.073	< 0.001	< 0.001
RNA5S4	-1.692	< 0.001	< 0.001	SPON2	-3.22	< 0.001	< 0.001
RNA5S5	-1.692	< 0.001	< 0.001	CD300LB	-3.146	< 0.001	< 0.001
RNA5S6	-1.692	< 0.001	< 0.001	CCR2	-2.201	< 0.001	< 0.001
RNA5S7	-1.692	< 0.001	< 0.001	ABHD15	-1.832	< 0.001	< 0.001
RNA5S8	-1.692	< 0.001	< 0.001	ZFP36L2	-1.464	< 0.001	< 0.001
CD300A	-2.557	< 0.001	< 0.001	PRICKLE3	-1.474	< 0.001	< 0.001
TGFBR3	-3.922	< 0.001	< 0.001	IFI44L	-3.043	< 0.001	< 0.001
CD99	-1.737	< 0.001	< 0.001	CCR5	-3.003	< 0.001	< 0.001
OAS1	-3.59	< 0.001	< 0.001	ID3	-1.874	< 0.001	< 0.001
MIR7641-2	-1.636	< 0.001	< 0.001	TXNDC5	-1.269	< 0.001	< 0.001
PLAC8	-1.626	< 0.001	< 0.001	RAC2	-1.244	< 0.001	< 0.001
KIAA0125	-2.059	< 0.001	< 0.001	CYTH4	-1.356	< 0.001	< 0.001
MMP23A	-3.094	< 0.001	< 0.001	TRAF3IP3	-1.442	< 0.001	< 0.001
GLIPR1	-1.619	< 0.001	< 0.001	PLCG2	-1.413	< 0.001	< 0.001
THBS1	-2.809	< 0.001	< 0.001	PROC	-1.653	< 0.001	< 0.001
FCER1A	-2.407	< 0.001	< 0.001	MMP23B	-2.807	< 0.001	< 0.001
CMTM3	-2.069	< 0.001	< 0.001	BLOC1S5-TXNDC5	-1.24	< 0.001	< 0.001
ATP13A2	-2.403	< 0.001	< 0.001	DUSP1	-1.689	< 0.001	< 0.001
DERL3	-1.505	< 0.001	< 0.001	CD52	-1.388	< 0.001	< 0.001
PECAM1	-2.112	< 0.001	< 0.001	APBB1IP	-1.265	< 0.001	< 0.001
C12orf75	-1.411	< 0.001	< 0.001	ANXA1	-1.885	< 0.001	< 0.001

Supplemental Table 3. Gene expression of markers related to AS-DCs and pDCs in control and TNF-treated cells.

		Raw Counts			Norm Counts			Differential Expression (TNF vs Control)		
Refseq Transcript	Gene Symbol	pDC1	pDC2	pDC3	pDC1	pDC2	pDC3	log2 Fold Change	*P*-Value	FDR
**AS-DC genes**										
NM_001177547	*SIGLEC6*	1	28	37	0.6	18.1	65.2	-0.836	0.189	NA
NM_001699	*AXL*	304	263	72	187.9	46.5	123.4	0.412	0.427	0.77
NM_023068	*SIGLEC1*	0	0	3	0	1.9	0	-0.004	0.991	NA
NM_001185100	*SIGLEC2*	56	61	1	34.6	0.6	7	0.888	0.184	0.53
NM_001772	*SIGLEC3*	67	0	143	41.4	92.4	1.8	-0.251	0.703	0.91
NM_004235	*KLF4*	160	20	40	98.9	25.8	128.6	-0.698	0.267	0.64
**pDC genes**										
NM_003199	*TCF4*	16602	21439	6601	10265.7	13848.4	11328.1	-0.412	0.065	0.33
NM_001244972	*NRP1*	217	656	179	134.2	423.7	315.5	-1.137	0.052	0.3
NM_016562	*TLR7*	1190	770	278	735.8	497.4	304.4	-0.491	0.208	0.57
NM_017442	*TLR9*	2035	3579	642	1258.3	2311.8	702.9	-0.981	0.002	0.05
